# ESPO AND NIA BUTLER-WILLIAMS SCIENTIFIC SYMPOSIUM: INFLUENCE OF RACE, ETHNICITY, AND EXPERIENCE ON HEALTH IN AGING

**DOI:** 10.1093/geroni/igz038.1281

**Published:** 2019-11-08

**Authors:** Jamie N Justice, Carl V Hill, Roland J Thorpe

**Affiliations:** 1 Wake Forest School of Medicine, Winston-Salem, North Carolina, United States; 2 NIH/National Institute on Aging, Bethesda, Maryland, United States; 3 Johns Hopkins Bloomberg School of Public Health, Baltimore, Maryland, United States

## Abstract

The NIA’s Butler-Williams Scholars Program and GSA’s Emerging Scholars and Professional Organization are united in providing career development opportunities for early career scholars in a manner that promotes leadership, diversity, and inclusivity. This provides a foundation to develop a network of next generation of scientists, clinicians, and policy makers capable of shaping health in aging. Among the chief concerns of our aging population are disparities in health associated with race/ethnicity, experience, environment, access to health care, and sociocultural and socioeconomic factors. GSA’s early career professionals and alumni of the prestigious NIA Butler-Williams Scholars Program have tackled these issues directly and the scientific scholarship that results is astounding in its breadth and depth. Dr. Vicki Johnson-Lawrence, Ph.D. (Butler-Williams class of 2014), will present on the interacting effects of education and race/ethnicity on multi-morbidity, highlighting lessons learned from the National Health Interview Study. Dr. Lauren Parker (Butler-Williams class of 2018) will review efforts to develop culturally competent content for recruitment of Hispanic and black/African American persons to NIA-supported dementia-caregiving studies. Dr. Ryon Cobb (Butler-Williams class of 2016) will discuss the impact of race/ethnicity on kidney function among older adults, with evidence from the Health and Retirement Study. The final speaker, Dr. Ana Quiñones (Butler-Williams class of 2012), will present on longitudinal tracking of multi-morbidity in racially/ethnically diverse older adults. In sum, the featured talks by rising stars in aging research deepen our understanding of the influence of race, ethnicity, and experience on health and chronic disease in diverse aging populations.

